# Quantitative Motion Analysis of Tai Chi Chuan: The Upper Extremity Movement

**DOI:** 10.1155/2018/2538765

**Published:** 2018-01-29

**Authors:** Tsung-Jung Ho, Kuei-Ting Chou, Cheng-Hsuan Li, Bor-Chen Kuo, Tzung-Chi Huang

**Affiliations:** ^1^School of Chinese Medicine, China Medical University, Taichung 40402, Taiwan; ^2^Division of Chinese Medicine, China Medical University Beigang Hospital, Yunlin 65152, Taiwan; ^3^Division of Chinese Medicine, An Nan Hospital, China Medical University, Tainan 70965, Taiwan; ^4^Department of Biomedical Imaging and Radiological Science, China Medical University, Taichung 40402, Taiwan; ^5^Graduate Institute of Educational Information and Measurement, National Taichung University of Education, Taichung 40306, Taiwan; ^6^Department of Artificial Intelligence in Medical Diagnosis, China Medical University Hospital, Taichung 41354, Taiwan

## Abstract

The quantitative and reproducible analysis of the standard body movement in Tai Chi Chuan (TCC) was performed in this study. We aimed to provide a reference of the upper extremities for standardizing TCC practice. Microsoft Kinect was used to record the motion during the practice of TCC. The preparation form and eight essential forms of TCC performed by an instructor and 101 practitioners were analyzed in this study. The instructor completed an entire TCC practice cycle and performed the cycle 12 times. An entire cycle of TCC was performed by practitioners and images were recorded for statistics analysis. The performance of the instructor showed high similarity (Pearson correlation coefficient (*r*) = 0.71 ~ 0.84) to the first practice cycle. Among the 9 forms, lay form had the highest similarity (*r*_mean_ = 0.90) and push form had the lowest similarity (*r*_mean_ = 0.52). For the practitioners, ward off form (*r*_mean_ = 0.51) and roll back form (*r*_mean_ = 0.45) had the highest similarity with moderate correlation. We used Microsoft Kinect to record the spatial coordinates of the upper extremity joints during the practice of TCC and the data to perform quantitative and qualitative analysis of the joint positions and elbow joint angle.

## 1. Introduction

 Tai Chi Chuan (TCC) is an internal Chinese martial art that focuses on integrating body movement, breath, and mind adjustments into one. The theory behind TCC is the manipulation of the proper flow of “*qi*” of the body to regulate bodily functions. Its gentle and slow movements make TCC very suitable for older people. Studies have shown that TCC practice in middle-aged and elderly adults benefits their body functions and disease prevention, including improvements of heart and lung functions [1, 2] and aerobic capacity [3], reducing the risks of cardiovascular diseases [4, 5], hypertension [6], rheumatoid arthritis [7], impaired immune system [8], and cancers [9, 10]. It can also reduce the incidences of depression and unease in the elderly and improve their quality of life [11–14]. In addition, long-term practice of TCC also helps to build stronger muscles and tendons [15, 16] and reduce bone density loss [17]. Studies have also shown that TCC can improve balance in the elderly [18–21]. In addition, another study that employed a molecular cellular approach showed that TCC has an antiaging effect [22].

Several biomechanical studies of TCC have been performed previously. Training in TCC includes the movement of* qi*, which requires “soft inside hard power and* Jin* inside soft power.” As the sequence of movements in the practice of TCC is very important, in order to provide students with a reference for the practice of TCC, Chen et al. [23] used several techniques to study the movement of the center gravity during TCC instruction. They recorded images during the practice of TCC by an instructor and analyzed the movement. Using surface electrodes to record muscle contractions and force plates to measure biophysical parameters, their study observed the shift of body weight during TCC [23]. On the other hand, Wu and coworkers [24] focused on the muscle action patterns during TCC exercise, including the tibialis anterior, soleus, peronaeus longus, rectus femoris, semitendinosus, and tensor fasciae latae. They analyzed the Tai Chi gait and its effect on balance, flexibility, and strength. In addition, Chua et al. [25] established a virtual environment that mimicked the practice of a TCC master, which can provide the learner with new ways to improve their skills. However, no study has been performed to date to conduct quantitative and reproducible analysis of the standard body movement in TCC, especially the upper extremities, which can be used as an accurate reference for TCC learners.

Precise extremity movement during the practice of TCC improves health, although there is still a lack of scientific analysis of body movement in TCC. In this study, we investigated the body movement in TCC (including the preparation form and eight essential forms: ward off, roll back, press, push, pluck, lay, elbow, and lean sideways) during the practice of a senior TCC master as well as TCC practitioners and quantitatively analyzed the movement patterns of their upper body. A Microsoft Kinect (MS Kinect) sensor was used to record images of movement, and skeleton tracking was employed to quantitatively analyze the reproducibility of the nine forms of TCC. The results of this study can be taken as a reference for standardizing TCC practice, and the quantitative analysis tools can be used in the process of qualification of TCC instructors and as the standard to examine the outcome of TCC practitioners.

## 2. Materials and Methods

### 2.1. Study Participant

101 TCC practitioners who had learned TCC for six months as a test group and a TCC master who is a doctor of traditional Chinese medicine, with more than 30 years of TCC practice, were included in this study. The instructor has extensive teaching experience in TCC and also taught at the Department of Traditional Chinese Medicine, China Medical University. He practices TCC three times a week and has done so for more than 30 years. He is also a national referee and national coach of TCC, certificated by National Tai Chi Chuan Association, Taiwan. A written informed consent has been obtained from each participant and the analysis performed in this study was approved by the Institutional Review Board of China Medical University Hospital (CMUH105-REC3-012).

### 2.2. Tai Chi Chuan (TCC)

**Figure 1:**
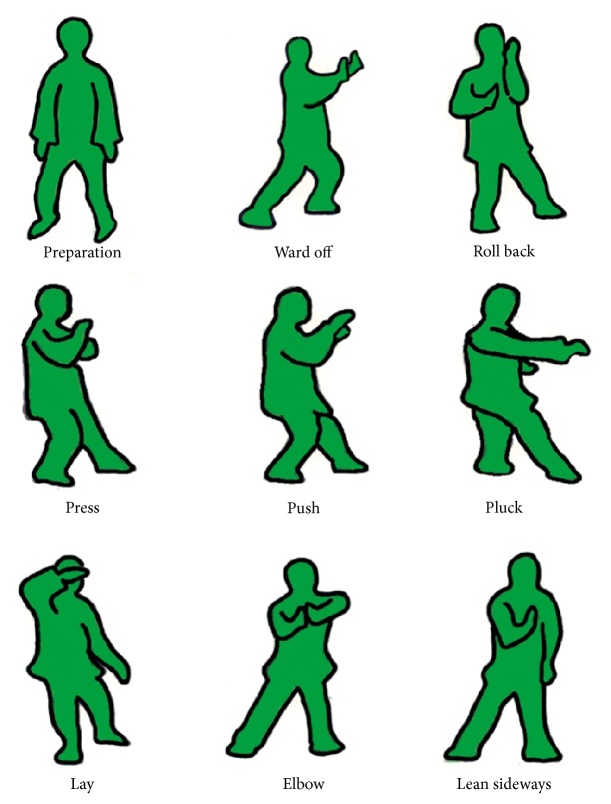
Illustration of the preparation form and the eight essentials of TCC (ward off, roll back, press, push, pluck, lay, elbow, and lean sideways).

The analysis of TCC in this study included examination of the complete practice cycle, which consists of the preparation form and eight other essentials of TCC. The eight essentials are ward off, roll back, press, push, pluck, lay, elbow, and lean sideways. The entire 9 forms are shown in Figure 1. The instructor completed an entire TCC practice cycle in 30 mins and performed the cycle 12 times. The first cycle of recorded movement was used as the reference in this study. And each practitioner performed the one time with the entire 9 forms of TCC.

### 2.3. Motion Data Collection

MS Kinect was used to record the motion of the instructor and practitioners during their TCC practices. To record an entire body image, the MS Kinect sensor was placed 4 meters in front of the subject on a tripod 80 cm above the ground. The image recording was set at 16 frames per second (fps).

**Figure 2:**
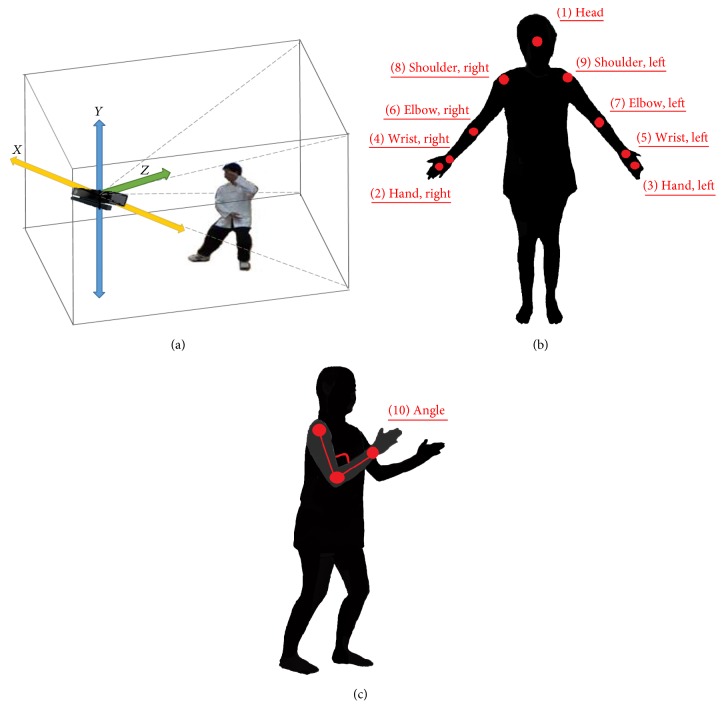
*Using MS Kinect for obtainment of skeleton tracking data*. (a) Definition of the individual *XYZ* directions. (b) The nine points included the head and the shoulders, elbows, wrists, and hands on both the left and right sides. (c) Calculation of the elbow joint angle.

MS Kinect enabled motion tracking to provide data for the calculation of skeleton joint coordinates [26], which revealed the 3D trajectories of the upper extremities during the practice of TCC by the instructor. As shown in Figure 2(a), the *XYZ* coordinates of each skeleton joint point indicated the distance of the point from the lens of the MS Kinect system (reference coordinate [0, 0, 0]). As illustrated in Figure 2(b), the coordinates of nine points, including the head and eight joint points (shoulders, elbows, wrists and hands on the left and right sides), were recorded by MS Kinect, and the data were used to calculate the angles of the upper extremities. Further analysis of the data indicated the changes in the elbow joint angles during the TCC of the instructor (Figure 2(c)).

### 2.4. Statistical Analysis

The coordinates of the joint points of the first cycle of the 12 practice cycles were used as the reference to analyze the similarity of the remaining 11 practice cycles and each practitioner cycle using Pearson correlation coefficient (*r*) measurement. The analysis calculated motion tracking of the joint points in each of the 9 TCC forms during the practice. The results are presented in bar chart form to show the similarity of each joint point and elbow angle between instructor's 12 practices, and the mean value (*r*_mean_) and standard deviation of the similarity for the first two forms with the highest and lowest similarities were calculated. Finally, the degree of similarity and average of similarity are presented using radar charts to evaluate the joint points and elbow joint angles with the greatest or least similarity.

## 3. Results

Ten of the 11 instructor's practice cycles had high similarity (*r* = 0.71 ~ 0.84) to the first practice cycle, while the third practice cycle had a lower similarity (*r*_mean_ = 0.64) to the reference (Table 1). Among the 9 forms, the lay form had the highest similarity (*r*_mean_ = 0.90) and the push form had the lowest similarity (*r*_mean_ = 0.52).

 The instructor performed 12 cycles of a complete TCC practice, and the first practice cycle was used as the reference. 1-2–1-12 indicate comparison of the 2nd–12th practice cycle to the first practice.

**Figure 3:**
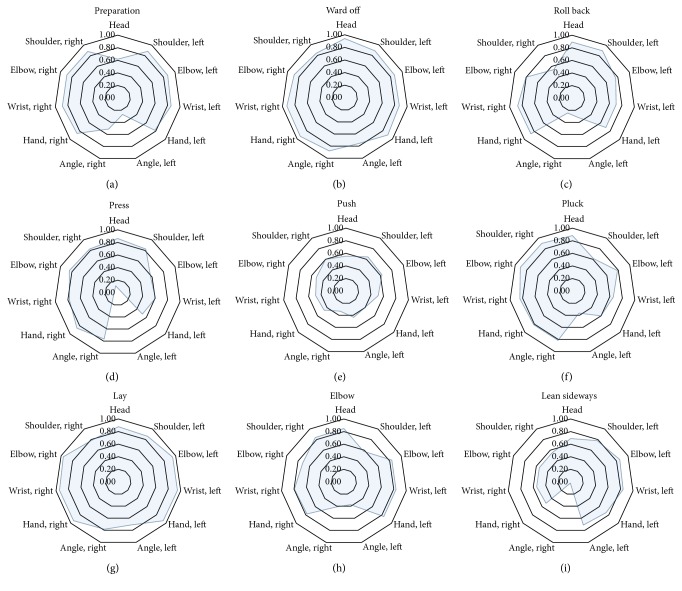
Average similarity values for each joint point and elbow joint angle were used to plot radar charts that show the dynamic similarity.

Using radar charts (Figure 3) to plot the similarity of the 11 practice cycles to the first cycle for all 9 forms, the dynamic similarity of each form in terms of each joint point and the elbow joint angle was observed, and the joint points with the greatest or lowest similarities were identified. Taking the push form and the lay form (the form of lowest and highest similarity, resp.) for example, in the push form, all the joint points and the elbow joint angles showed moderate correlation in terms of dynamic similarity (Figure 3(e)), the poorest being the right elbow joint angle (*r*_mean_ = 0.33). Among the 12 forms, the lay form was the form with the highest dynamic similarity, with all joint points and elbow joint angles exhibiting high correlation (Figure 3(g)).

**Figure 4:**
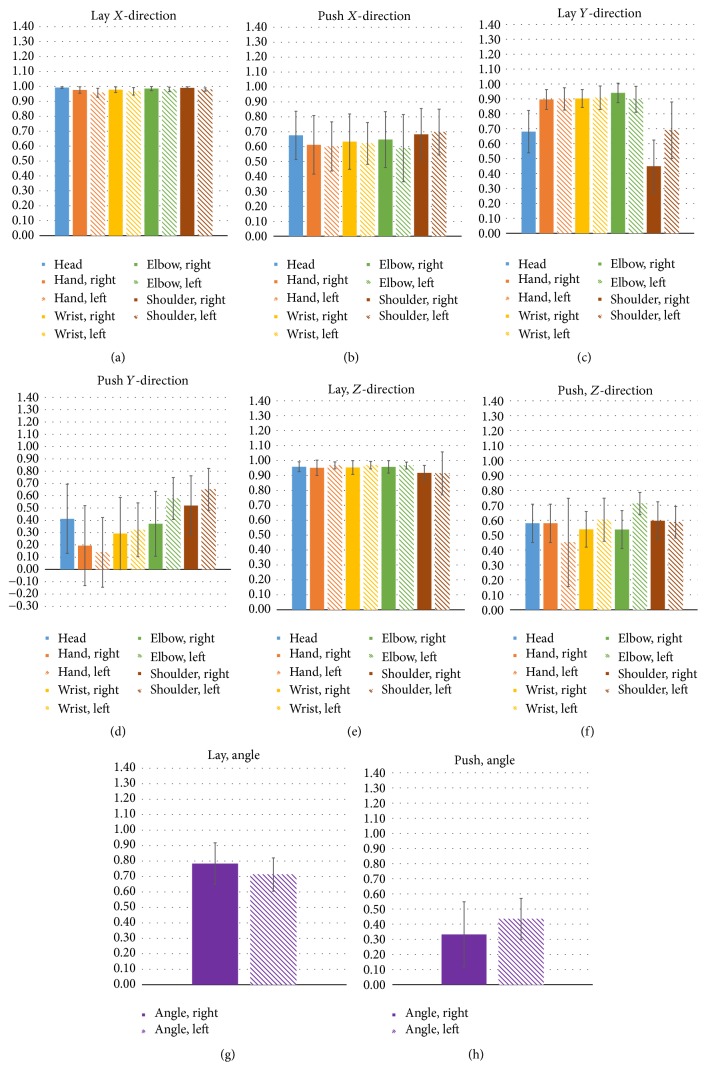
Comparison of the similarity of all joint points in the *X*-, *Y*-, and *Z*-directions and elbow joint angles in the practice of the lay and push forms.

Figure 4 shows a comparison of the similarity of the first practice cycle with the remaining 11 practice cycles for the forms of the highest and lowest similarity (lay and push forms, resp.). The bar charts demonstrate the similarity of each joint point in the *X*-, *Y*-, and *Z*-coordinates and the elbow joint angles. The *X*-direction (right-left or lateral direction) and the similarities of all joints in the lay form were close to 1 (Figure 4(a)), while only the left shoulder had a higher similarity (*r*_mean_ = 0.7) in the push form, and the remaining points had only a moderate correlation (Figure 3(b)). In the *Y*-direction (head-feet or vertical direction), the right shoulder had a poor correlation (*r*_mean_ = 0.45), and the head (*r*_mean_ = 0.68) and left shoulder (*r*_mean_ = 0.68) had moderate correlations in the lay form (Figure 4(c)); all points had moderate or poor correlation in the push form, the point with the poorest correlation (the hand) having a mean correlation value of 0.14 (Figure 4(d)). In the *Z*-direction (distance to the lens), all points showed a high correlation, with *r* values higher than 0.9 in the lay form (Figure 4(e)); in the push form, only the left elbow showed a high correlation, with an *r* value of 0.71, while the remaining points showed only moderate correlation (Figure 4(f)). In terms of the elbow joint angles, both the left and right elbow joint angles showed high correlation in the lay form; only moderate correlation was seen for the left and right elbow joint angles in the push form (Figure 4(h)).

The average similarity of 101 TCC practitioners' practice cycle and each joint point is shown in Table 2. Among the 9 forms, the ward off form and the roll back form had moderate correlation (*r*_mean_ = 0.51, 0.45), and remaining form had low correlation. Elbow joint angles had the lowest correlation of all joint point (*r*_mean_ = 0.17).

The TCC practitioners performed 1 cycle of a complete TCC practice, and the first practice cycle of instructors was used as the reference.

## 4. Discussion

Table 1 demonstrates the similarity of the 11 practice cycles of each TCC form when the first practice cycle was used as the standard. Among the nine forms, the lay form and the push form had the highest and lowest similarity, respectively. The radar chart (Figure 3(e)) also showed that all joint points had poor dynamic similarity in the push form. And as shown in the bar charts presented in Figure 4, the *XYZ* coordinates and elbow joint angles showed that the push form, with the poorest performance, had a low similarity in the *Y*-direction, and both the left and right hand joints were the weak points. The push form concentrates on movement of the hands, requiring turning the palms over, moving them close to the chest, and then pushing them out. Although the push form is relatively easier to attain than other forms, significant error is easily created, as the hands need to perform precise movements in a small space. As shown in Figure 4(b), the results indicated that both the left and right hands had poor dynamic similarity with greater standard deviation in the push form, and the remaining joint points also had lower dynamic similarity. In addition, even in the lay form, which had the highest dynamic similarity, the head and both shoulder joint points had only moderate correlation in the *Y*-direction. The results suggested that learners should pay particular attention to accuracy of posture, especially in the *Y*-direction for joint points of the upper extremities. Moreover, further comparison of the right and left hands (dominant hand and nondominant hand, resp.) demonstrated that there was no significant difference between the right and left hand (*p* > 0.05).

**Figure 5:**
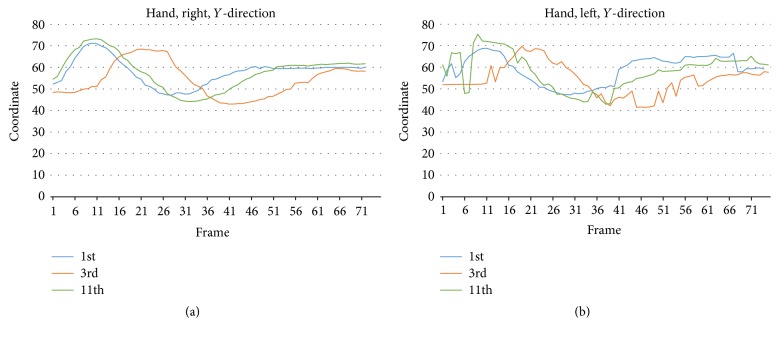
Comparison of the practice cycles with the highest (11th cycle) and lowest (3rd cycle) dynamic similarity to the reference cycle (1st cycle). The plots reveal the movement of (a) the right and (b) the left hand in the *Y*-direction.

Next, we carried out a detailed analysis of the 12 practice cycles of the push form, which focused on the movement in the *Y*-direction of the two joint points (left and right hands) with the poorest performance. The analysis indicated that the 11th practice cycle had the highest dynamic similarity, the right and left hands having correlation values of 0.90 and 0.72, respectively; the 3rd practice cycle had the poorest dynamic similarity, the right and left hands having correlation values of only −0.23 and −0.22, respectively. As shown in Figure 5, the left and right hands exhibited slow movement in the beginning, which caused poor dynamic similarity. If the movement of 3rd cycle could start a second earlier, the similarity would be much better. TCC learners may use this qualitative and quantitative analysis method to improve the accuracy of their TCC practice.

We also evaluated the TCC practitioners' practice with dynamic similarity (Table 2). Among all forms, the ward off form and the Roll back form had higher dynamic similarity than other forms, which can be explained by the fact that the range of movement of these two forms is large so that it is easier to learn for learners. The dynamic similarity of elbow joint angle was significantly lower than other joint points. Because all students practice TCC twice a week and only for six months, they were not very skillful. Based on the analysis, instructor should put emphasis on the continuity of movement when he is teaching TCC to beginners.

In previous studies, Wu and colleagues [24] used two biomechanical force plates and a marker-based motion analysis system equipped with 3 camera configurations to track reflective markers attached to test subjects (at the fifth metatarsal head, heel, lateral malleolus, femoral epicondyle, greater trochanter, mid-leg, mid-thigh, and shoulder). They also included electromyography measurement to record signals of leg muscles and stomach muscles during the practice of their test subjects and performed quantitative comparisons. Although a system with biomechanical force plates may effectively measure leg motion in the gait cycle, it requires bony landmarks with multiple cameras that use reflective markers along with anthropometrical measurements to compute the positions of joints. The process is complicated and requires an expensive device for the experiment. In contrast, we demonstrated that the skeleton tracking function of MS Kinect can accurately and easily be used to perform dynamic analysis of the joint points of the upper extremities. In addition, using skeleton tracking to identify the coordinates of joints for dynamic analysis has the advantage of being low in cost and more widely available than motion analysis systems incorporating several cameras.

Chua and coworkers [25] used a Vicon real-time optical motion capture system that provided one virtual environment to evaluate the training outcome for TCC beginners. However, unlike our study, which investigated the preparation form and the eight essentials of TCC, their study only focused on body movement in TCC practice, which just puts the focus on the beginner mimicking the teacher's movement. In addition, the Vicon real-time optical motion capture system is more difficult to install and more expensive than MS Kinect and thus is only accessible to wealthy laboratories and is not suitable for family use. Our current study employed MS Kinect to record the spatial coordinates of the joints of the upper extremities and then performed qualitative and quantitative analysis of body movement. The qualitative and quantitative analysis provided radar charts that revealed the dynamic similarity of each practice cycle with the first reference cycle, which would allow the learner to identify the joint points with an excellent or poor performance. The results can be divided into *X*-, *Y*-, and *Z*-directions, which provides information to the learner to improve their practice to the highest standard.

One of the limitations was the sample size. Validating the sample size could enhance the generalization of the study. Gender difference or years of chronic diseases might be one of the important influencing factors affecting the joint positions, as well as increasing the depression rate. Besides, this study only focused on the movements of the upper extremities. Future study will extend this analysis to lower extremity movements, body rotation, and gravity position change in order to cover the entire body movement, which will render this assessment method more complete. Increase of the sample size for gender difference and years of chronic diseases affecting the joint positions during Tai Chi Chuan will be also included.

In addition, this scientific method can be used to establish a standard for TCC practice. We will also employ this approach to study TCC learners of different age groups, different populations, or under different training methods in order to examine the dynamic similarity to assess the training outcome.

## 5. Conclusions

In this study, we successfully used a MS Kinect sensor to record the spatial coordinates of the upper extremity joints during the practice of a senior TCC master and used the data to perform quantitative and qualitative analysis of the joint positions and elbow joint angle. By analyzing the dynamic similarity of each practice cycle and each TCC form, this approach can be used to establish a system to set the standard of TCC practice. From the results presented by radar chart, the joint points with the best and worst performance can be identified, and the learner would be able to use the outcome to assess their weak points. Use of this MS Kinect application for evaluation of body movement in the practice of TCC will be of great value for those undergoing TCC training.

## Figures and Tables

**Table 1:** Comparison of the similarity of the nine TCC forms.

	1-2	1-3	1-4	1-5	1-6	1-7	1-8	1-9	1-10	1-11	1-12	*r*_mean_
Preparation	0.65	0.50	0.82	0.89	0.79	0.87	0.85	0.84	0.91	0.85	0.89	0.81
Ward off	0.90	0.38	0.95	0.92	0.96	0.96	0.98	0.94	0.94	0.93	0.94	0.89
Roll back	0.74	0.61	0.65	0.69	0.90	0.74	0.76	0.71	0.84	0.69	0.91	0.75
Press	0.60	0.81	0.75	0.74	0.63	0.77	0.54	0.79	0.79	0.83	0.60	0.71
Push	0.53	0.62	0.39	0.62	0.53	0.66	0.58	0.41	0.27	0.88	0.28	0.52
Pluck	0.61	0.69	0.77	0.72	0.81	0.84	0.79	0.76	0.87	0.75	0.80	0.77
Lay	0.93	0.88	0.94	0.91	0.90	0.87	0.78	0.91	0.94	0.94	0.91	0.90
Elbow	0.70	0.73	0.72	0.74	0.74	0.84	0.86	0.85	0.78	0.84	0.54	0.76
Lean sideways	0.72	0.55	0.72	0.53	0.37	0.62	0.77	0.78	0.83	0.79	0.51	0.65
*r*_mean_	0.71	0.64	0.74	0.75	0.74	0.80	0.77	0.78	0.80	0.84	0.71	

**Table 2:** Comparison of the average similarity of the nine TCC forms and joint points.

	Head	Shoulder		Elbow		Wrist		Hand		Angle		*r*_mean_
		Left	Right	Left	Right	Left	Right	Left	Right	Left	Right	
Preparation	0.30	0.32	0.32	0.32	0.31	0.32	0.29	0.31	0.29	0.21	0.19	0.29
Ward off	0.56	0.62	0.51	0.57	0.49	0.61	0.52	0.46	0.50	0.46	0.34	0.51
Roll back	0.55	0.52	0.58	0.52	0.55	0.55	0.51	0.39	0.53	0.11	0.13	0.45
Press	0.44	0.42	0.43	0.44	0.41	0.44	0.39	0.41	0.43	0.10	0.09	0.37
Push	0.44	0.33	0.36	0.35	0.41	0.40	0.40	0.44	0.44	0.12	0.14	0.35
Pluck	0.42	0.40	0.41	0.38	0.42	0.34	0.44	0.44	0.43	0.32	0.30	0.39
Lay	0.38	0.38	0.36	0.37	0.37	0.40	0.38	0.34	0.39	0.12	0.14	0.33
Elbow	0.41	0.37	0.41	0.33	0.41	0.34	0.37	0.37	0.41	0.09	0.10	0.33
Lean sideways	0.43	0.38	0.28	0.40	0.28	0.38	0.31	0.32	0.37	0.02	0.02	0.29

*r*_mean_	0.44	0.41	0.41	0.41	0.40	0.42	0.40	0.39	0.42	0.17	0.16	
		0.41		0.41		0.41		0.40		0.17		

## References

[1] Lan C., Lai J.-S., Wong M.-K., Yu M.-L. (1996). Cardiorespiratory function, flexibility, and body composition among geriatric Tai Chi Chuan practitioners. *Archives of Physical Medicine and Rehabilitation*.

[2] Zheng G., Li S., Huang M., Liu F., Tao J., Chen L. (2015). The effect of Tai Chi training on cardiorespiratory fitness in healthy adults: a systematic review and meta-analysis. *PLoS ONE*.

[3] Taylor-Piliae R. E., Froelicher E. S. (2004). The effectiveness of Tai Chi exercise in improving aerobic capacity: a meta‐analysis. *Journal of Cardiovascular Nursing*.

[4] Cheng T. O. (2007). Tai Chi: The Chinese ancient wisdom of an ideal exercise for cardiac patients. *International Journal of Cardiology*.

[5] Lee M. S., Pittler M. H., Taylor-Piliae R. E., Ernst E. (2007). Tai chi for cardiovascular disease and its risk factors: a systematic review [1]. *Journal of Hypertension*.

[6] Lee M. S., Pittler M. H., Guo R., Ernst E. (2007). Qigong for hypertension: a systematic review of randomized clinical trials. *Journal of Hypertension*.

[7] Lee M. S., Pittler M. H., Ernst E. (2007). Tai chi for rheumatoid arthritis: systematic review. *Rheumatology*.

[8] Irwin M., Pike J., Oxman M. (2004). Shingles Immunity and Health Functioning in the Elderly:. *Evidence-Based Complementary and Alternative Medicine*.

[9] Lee M. S., Chen K. W., Sancier K. M., Ernst E. (2007). Qigong for cancer treatment: a systematic review of controlled clinical trials. *Acta Oncologica*.

[10] Lee M. S., Pittler M. H., Ernst E. (2007). Is Tai Chi an effective adjunct in cancer care? a systematic review of controlled clinical trials. *Supportive Care in Cancer*.

[11] Ho T.-J., Wen-Miin L., Lien C.-H. (2007). Health-related quality of life in the elderly practicing T'ai Chi Chuan. *The Journal of Alternative and Complementary Medicine*.

[12] Jahnke R., Larkey L., Rogers C., Etnier J., Lin F. (2010). A comprehensive review of health benefits of qigong and tai chi. *American Journal of Health Promotion*.

[13] Rogers C. E., Larkey L. K., Keller C. (2009). A review of clinical trials of tai chi and qigong in older adults. *Western Journal of Nursing Research*.

[14] Verhagen A. P., Immink M., van der Meulen A., Bierma-Zeinstra S. M. A. (2004). The efficacy of Tai Chi Chuan in older adults: a systematic review. *Journal of Family Practice*.

[15] Lan C., Lai J.-S., Chen S.-Y., Wong M.-K. (2000). Tai Chi Chuan to improve muscular strength and endurance in elderly individuals: A pilot study. *Archives of Physical Medicine and Rehabilitation*.

[16] Wu G., Zhao F., Zhou X., Wei L. (2002). Improvement of isokinetic knee extensor strength and reduction of postural sway in the elderly from long-term Tai Chi exercise. *Archives of Physical Medicine and Rehabilitation*.

[17] Woo J., Hong A., Lau E., Lynn H. (2007). A randomised controlled trial of Tai Chi and resistance exercise on bone health, muscle strength and balance in community-living elderly people. *Age and Ageing*.

[18] Ho T., Chen S., Hong S., Lu T., Lin J. (2012). Influence of long-term Tai-Chi Chuan training on standing balance in the elderly. *Biomedical Engineering : Applications, Basis, and Communications*.

[19] Tsang W. W. N., Hui-Chan C. W. Y. (2004). Effect of 4- and 8-wk intensive tai chi training on balance control in the elderly. *Medicine & Science in Sports & Exercise*.

[20] Dikmen S. S., Bombardier C. H., Machamer J. E., Fann J. R., Temkin N. R. (2004). Natural history of depression in traumatic brain injury. *Archives of Physical Medicine and Rehabilitation*.

[21] Wong A. M., Lin Y.-C., Chou S.-W., Tang F.-T., Wong P.-Y. (2001). Coordination exercise and postural stability in elderly people: effect of Tai Chi Chuan. *Archives of Physical Medicine and Rehabilitation*.

[22] Ho T.-J., Ho L.-I., Hsueh K.-W. (2014). Tai Chi intervention increases progenitor CD34^+^ cells in young adults. *Cell Transplantation*.

[23] Chen H.-C., Cheng K.-Y. B., Liu Y.-J., Chiu H.-T., Cheng K.-Y. (2010). The defence technique in Tai Chi Push Hands: A case study. *Journal of Sports Sciences*.

[24] Wu G., Liu W., Hitt J., Millon D. (2004). Spatial, temporal and muscle action patterns of Tai Chi gait. *Journal of Electromyography & Kinesiology*.

[25] Chua P. T., Crivella R., Daly B. Training for physical tasks in virtual environments: Tai Chi.

[26] Zhang Z. (2012). Microsoft kinect sensor and its effect. *IEEE MultiMedia*.

